# Gastric cancer incidence in the Faroe Islands.

**DOI:** 10.1038/bjc.1984.166

**Published:** 1984-08

**Authors:** J. T. Poulsen, O. M. Jensen, M. Egholm, K. Hou-Jensen


*Br. J. Cancer* (1984), **50**, 223–225

### Short Communication

### Gastric cancer incidence in the Faroe Islands

J. Thomas Poulsen[1], O. Møller Jensen[2], M. Egholm[3] & K. Hou-Jensen[1]

[1]*Department of Pathology, Finsen Institute;* [2]*Danish Cancer Registry, Copenhagen, Denmark;* [3]*Department of Pathology, The Regional Hospital, Thorshavn, Faroe Islands.*

Gastric cancer is still frequent in many countries (Waterhouse *et al.*, 1982), although a worldwide downward trend in the incidence has been observed during the last three decades. Among the Nordic countries, Iceland and Finland are high risk areas, while Denmark and Sweden are low risk areas (Jensen, 1982).

The aetiology of gastric cancer is largely unknown, but several factors of a dietary nature have been suggested (Tulinius, 1978).

Significant difference between the Faroese and the high Icelandic gastric cancer incidence rates may serve as a basis for illuminating the aetiology of gastric cancer.

The Faroes are a group of 18 islands situated in the North Atlantic. Since 1948 the Faroe Islands have home rule within the Danish state. The Faroese are of Scandinavian origin. The original settlers came from Norway in the 9th century, but admixture of a Celtic strain has been confirmed by genetic markers, e.g. ABO-blood-groups (Mourant *et al.*, 1976). During the period of current interest from 1965 to 1981 the Faroese population increased from some 37,000 to 44,000 inhabitants. Fish and fishproducts total 90% of the exports from the Faroe Islands.

There are 3 general hospitals in the Faroes with 358 beds and 41 hospital doctors, among them specialists in gastroenterology, surgery, X-ray diagnosis and pathology.

All new cases of gastric cancer (ICD-151) during the period 1965-1981 were identified by examination of the records of the 3 Faroese hospitals and the files of a local cancer registry operating in the regional hospital in Thorshavn during the years 1965–1972. In addition all death certificates from the same period were examined together with all reports from Danish hospitals to the Danish Cancer Registry on persons residing in the Faroe Islands.

For the purpose of incidence computations each patient was included only once, when multiple source information was available. Only gastric cancer cases among persons residing in the Faroe Islands at the time of diagnosis were included in the study.

The average of the age- and sex-specific populations of the 3 censuses in the Faroes in 1966, 1970, and 1977 (Danmarks Statistik, 1979) were used as denominators for the calculation of the incidence rates (Table I). Some 98 cases among men and 49 cases among women of newly diagnosed gastric cancer were identified in the Faroese population during the 17 years from 1965 to 1981. Three cases (2%) were included with information from the death certificate only. Six cases (4%) were diagnosed by clinical examination alone, 13 cases (9%) verified by X-ray, and 12 cases (8%) by laparotomy without biopsy. In 72 cases (49%) the diagnosis was verified by gastric resection and subsequent histopathological examination. In the remaining 41 cases (28%) the diagnosis was verified by gastroscopic examination with biopsy.

During the whole period some 74.8% of the cases were histologically verified (men 77.0%, women

**Table I** Age specific, crude and age-standardized annual incidence rates per 100,000 of gastric cancer (ICD 151) in the Faroe Islands, 1965–1981.

| *Age groups* | *No. of cases 1965–1981* | | *Annual incidence per 100,000* | |
|---|---|---|---|---|
| | Males | Females | Males | Females |
| 0–34 | 0 | 0 | 0.0 | 0.0 |
| 35–39 | 1 | 0 | 5.0 | 0.0 |
| 40–44 | 0 | 1 | 0.0 | 5.9 |
| 45–49 | 0 | 3 | 0.0 | 17.2 |
| 50–54 | 4 | 3 | 22.6 | 17.9 |
| 55–59 | 7 | 3 | 42.2 | 20.2 |
| 60–64 | 17 | 8 | 122.8 | 64.0 |
| 65–69 | 24 | 6 | 219.1 | 58.8 |
| 70–74 | 18 | 6 | 236.9 | 71.0 |
| 75–79 | 16 | 8 | 296.0 | 131.5 |
| 80–84 | 8 | 9 | 261.9 | 226.6 |
| 85– | 3 | 2 | 175.3 | 88.2 |
| All ages | 98 | 49 | 28.1 | 15.4 |
| Age-standardized: | | | | |
| World population | | | 24.5 | 11.6 |
| Truncated (35–64 years) | | | 25.8 | 17.9 |

Correspondence: J.T. Poulsen, Department of Pathology, Finsen Institute, Strandboulevarden 49, DK-2100 Copenhagen ø.
Received 11 March 1984; accepted 13 April 1984.

© The Macmillian Press Ltd., 1984

69.4%). An increasing proportion of histological verification from 62.1% in 1965–1972 to 85.2% in 1973–1981 was noted.

The age distribution of gastric cancer cases as well as incidence rates are given in Table I. The crude annual incidence rate is 28.1 cases per 100,000 men and 15.4 cases per 100,000 women. For the sake of comparison (Table II), age-standardized rates (world population) have been calculated for all ages and truncated for the age-groups 35–64.

The tumour localization was specified in 128/147 cases with the following distribution in the stomach: antrum 40.6%, corpus 36.8%, and cardia and fundus 22.6%.

The Faroese age-specific incidence rates run at a higher level then the Danish ones (Danish Cancer Registry, 1982), but with a drop in the oldest age-groups (Table I), most probably due to underdiagnosis, but random fluctuatuons are also likely to occur in the old age-groups with so few individuals. Apart from this the age-specific incidence pattern and sex ratio of ~2 are similar to those in Denmark.

**Table II** The age-standardized annual incidence per 100,000 of gastric cancer (ICD 151) in the Nordic countries (world standard population).

| *Country and time period* | *Incidence per 100,000* | | *Truncated incidence per 100,000 (35–64 y)* | |
|---|---|---|---|---|
| | *M* | *F* | *M* | *F* |
| Iceland | | | | |
| 1965–71 | 43.6 | 19.0 | 52.8 | 14.5 |
| 1972–77 | 36.6 | 16.7 | 44.8 | 20.3 |
| Finland | | | | |
| 1965–71 | 37.7 | 19.3 | 46.8 | 21.0 |
| 1972–76 | 29.5 | 15.0 | 36.0 | 17.5 |
| Norway | | | | |
| 1965–71 | 27.3 | 14.6 | 32.6 | 16.0 |
| 1972–74 | 21.1 | 10.9 | 26.3 | 14.0 |
| Sweden | | | | |
| 1965–71 | 21.2 | 11.3 | 22.6 | 12.5 |
| 1972–75 | 18.1 | 9.4 | 19.5 | 10.4 |
| Denmark | | | | |
| 1965–71 | 21.4 | 11.7 | 23.1 | 11.1 |
| 1972–77 | 16.7 | 9.0 | 18.1 | 9.6 |
| Faroe Islands | | | | |
| 1965–73 | 25.5 | 11.9 | 28.7 | 18.3 |
| 1974–81 | 22.3 | 11.3 | 21.8 | 17.1 |

To minimize the effect of possible underdiagnosis in the older age groups, the most valid comparisons should be based on the truncated rates.

When the two periods of 1965–1973 and 1974–1981 are compared we find a downward trend in the age-standardized truncated rates (35–64 years), (Table II), which seem to be less pronounced than in the remainder ot the Nordic region.

Our findings corroborate the trend in gastric cancer mortality in the Faroes (Niclasen, 1966) and indicate that the worldwide downward trend in the incidence of gastric cancer is also apparent in the Faroes during the last 25 years.

Although Iceland and the Faroe Islands have much in common both geographically, culturally, and genetically the present study indicates that the Faroese incidence of gastric cancer is much closer to the low Danish rates than to the high Icelandic rates, at least for males, (Table II). This is surprising, considering that high incidence of gastric cancer is usually connected with island realms (e.g. Japan, Iceland) with a high fish and salt consumption (Tulinius, 1978; Joossens & Geboers, 1981). Traditionally there is a high consumption of smoked food stuff in Iceland, and this dietary habit has been linked aetiologically with the high gastric cancer incidence in Iceland. Dungal & Sigurjonsson (1967) found a positive correlation between the consumption of smoked food and the mortality from gastric cancer in different places in Iceland, and Choi *et al.* (1971) found the same correlation among first and second generation Icelanders who had migrated to Canada. In the Faroes the consumption of fish and mutton is high as in Iceland, but apart from singed sheepheads there is not tradition for smoked food in the Faroe Islands.

These difference in dietary habits seem to offer yet a further indication that the consumption of smoked foodstuffs may play a part in the development of stomach cancer. The observed differences in the gastric cancer incidence in two closely related North Atlantic populations may form the basis for further investigations of the aetiological factors in gastric cancer.

This study was supported by grants from the Danish Hospital Foundation for Medical Research. Region of Copenhagen, the Faroe Islands, and Greenland. (J. no. 45/81).

**References**

CHOI, N.W., ENTWISTLE, D.W., MICHALUK, W. & NELSON, L. (1971). Gastric cancer in Icelanders in Manitoba. *Israel J. Med. Sci.*, **7**, 1500.

DANISH CANCER REGISTRY. (1982). *Incidence of Cancer in Denmark 1973–77.* Danish Cancer Registry, Danish Cancer Society, Copenhagen.

DANMARKS STATISTIK. (1970 I, 1975 I, 1979 VII). Statistisk tabelværk. København.

DUNGAL, N. & SIGURJONSSON, J. (1967). Gastric cancer and diet. *Br. J. Cancer*, **21**, 270.

JENSEN, O.M. (1982). Trends in the incidence of stomach cancer in the five Nordic countries. In: *Trends in Cancer Incidence. Causes and Practical Implications.* (Ed. Magnus) New York: Hemisphere Publication Corporation.

JOOSSENS, J.V. & GEBOERS, J. (1981). Nutrition and gastric cancer. *Proc. Nutr. Soc.*, **40**, 37.

MOURANT, A.D., KOPEC, A. & DOMANIEWSKA-SOBEZAK, K. (Ed.) 1976). *The distribution of the Human Blood Groups,* 2nd edition. London: Oxford University press.

NICLASEN, S.D. (1966). Cancer mortality in the Faroe Islands 1955–59. *Fródskaparrit,* **15**, 113.

TULINIUS, H. (1978). Epidemiology of gastric cancer. Näringsforskning. Supplementum, **16**, 53.

WATERHOUSE, J., MUIR, C., SHANMUGARATNAM, K. & POWELL, J. (Ed.) (1982). *Cancer Incidence in Five Continents. Vol. IV.* Lyon: IARC Scientific Publications, No. 15.


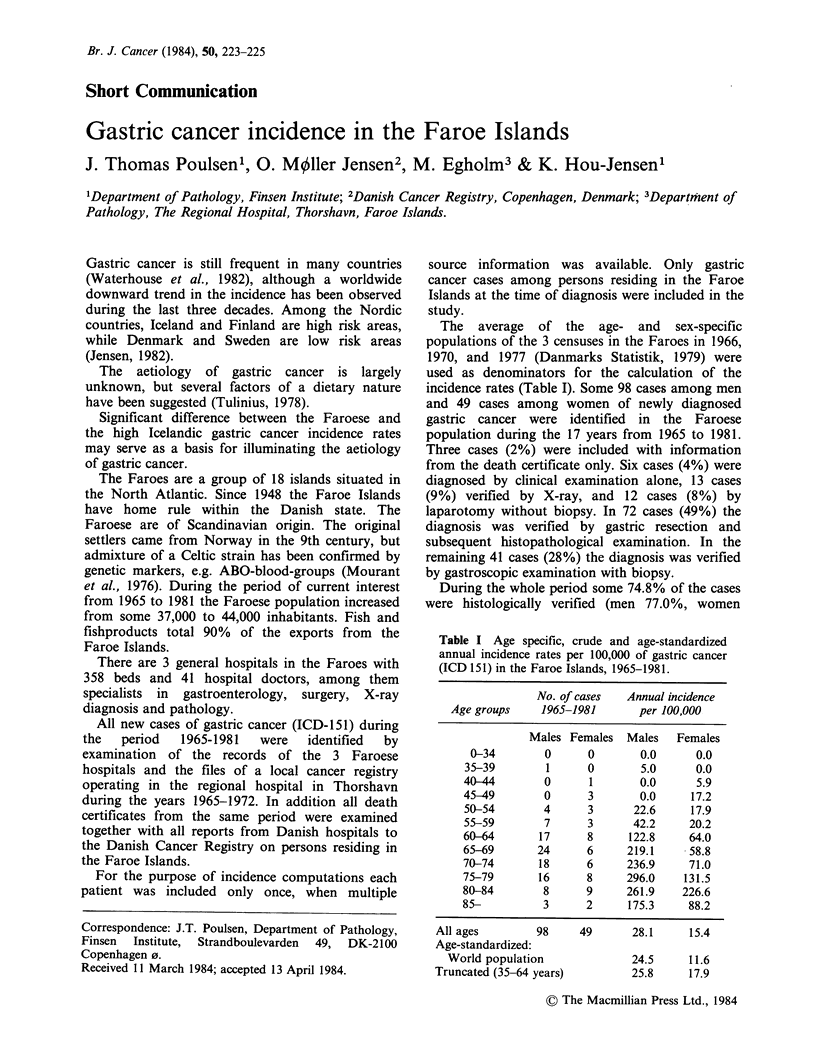

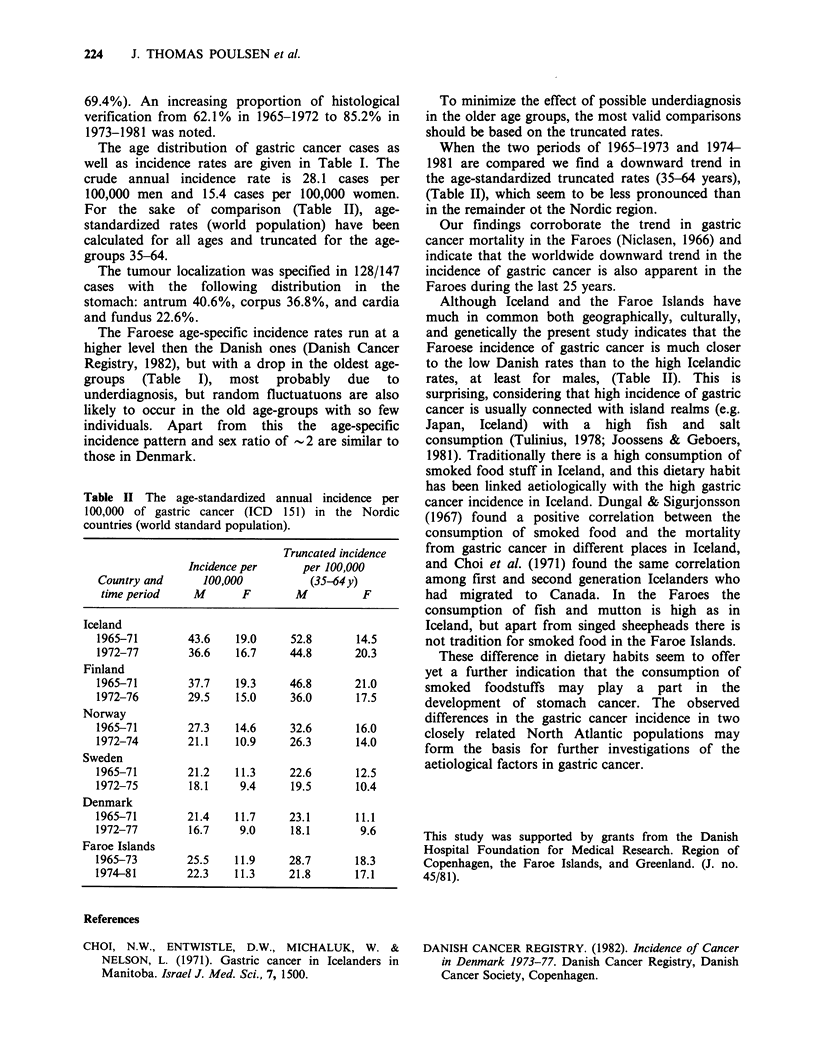

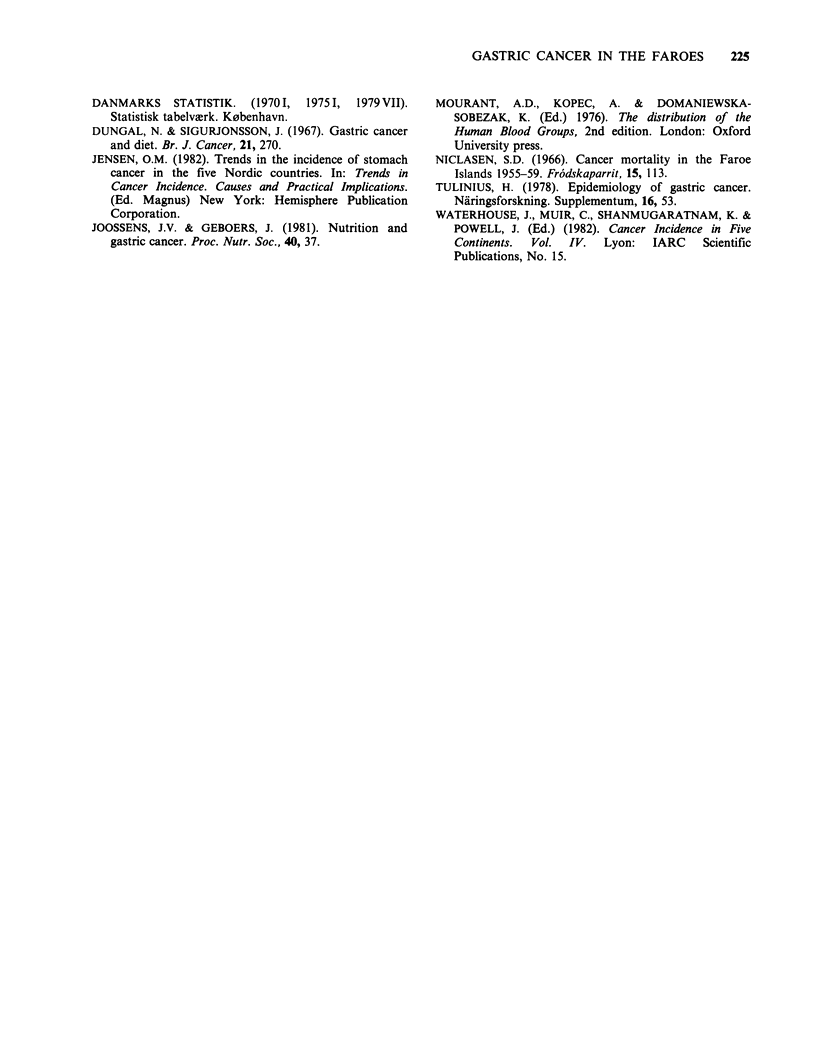


## References

[OCR_00251] Choi N. W., Entwistle D. W., Michaluk W., Nelson N. (1971). Gastric cancer in Icelanders in Manitoba.. Isr J Med Sci.

[OCR_00267] Dungal N., Sigurjonsson J. (1967). Gastric cancer and diet. A pilot study on dietary habits in two districts differing markedly in respect of mortality from gastric cancer.. Br J Cancer.

[OCR_00278] Joossens J. V., Geboers J. (1981). Nutrition and gastric cancer.. Proc Nutr Soc.

